# Toward improved understanding of foot shape, foot posture, and foot biomechanics during running: A narrative review

**DOI:** 10.3389/fphys.2022.1062598

**Published:** 2022-12-08

**Authors:** Qichang Mei, Hyun Kyung Kim, Liangliang Xiang, Vickie Shim, Alan Wang, Julien S. Baker, Yaodong Gu, Justin Fernandez

**Affiliations:** ^1^ Faculty of Sports Science, Ningbo University, Ningbo, China; ^2^ Research Academy of Grand Health, Ningbo University, Ningbo, China; ^3^ Auckland Bioengineering Institute, The University of Auckland, Auckland, New Zealand; ^4^ School of Kinesiology, Louisiana State University, Baton Rouge, LA, United States; ^5^ Centre for Health and Exercise Science Research, Hong Kong Baptist University, Kowloon, Hong Kong SAR, China; ^6^ Department of Engineering Science, The University of Auckland, Auckland, New Zealand

**Keywords:** foot shape, foot posture, statistical shape modeling, principal component analysis, foot biomechanics

## Abstract

The current narrative review has explored known associations between foot shape, foot posture, and foot conditions during running. The artificial intelligence was found to be a useful metric of foot posture but was less useful in developing and obese individuals. Care should be taken when using the foot posture index to associate pronation with injury risk, and the Achilles tendon and longitudinal arch angles are required to elucidate the risk. The statistical shape modeling (SSM) may derive learnt information from population-based inference and fill in missing data from personalized information. Bone shapes and tissue morphology have been associated with pathology, gender, age, and height and may develop rapid population-specific foot classifiers. Based on this review, future studies are suggested for 1) tracking the internal multi-segmental foot motion and mapping the biplanar 2D motion to 3D shape motion using the SSM; 2) implementing multivariate machine learning or convolutional neural network to address nonlinear correlations in foot mechanics with shape or posture; 3) standardizing wearable data for rapid prediction of instant mechanics, load accumulation, injury risks and adaptation in foot tissue and bones, and correlation with shapes; 4) analyzing dynamic shape and posture *via* marker-less and real-time techniques under real-life scenarios for precise evaluation of clinical foot conditions and performance-fit footwear development.

## Highlights

• The artificial intelligence is a useful metric of foot posture, which can be measured quickly and associated with motion and joint pain in adults, but may be less useful in special populations such as developing and obese individuals.
• The foot posture index that is used for classifying feet as pronated, neutral, and supinated is a useful approach under both static and dynamic conditions. However, care must be taken to associate pronation with injury risk, and factors of the Achilles tendon and longitudinal arch angles may be required for elucidating the risks.
• Dynamic tracking of foot shape, posture, and internal multi-segmental motion should consider current fluoroscopy, machine learning, statistical shape modeling, wearable, and marker-less techniques.

## Introduction

Changes of shape and posture in the human foot have been informed through the evolutionary process (Bennett et al., 2009; Lieberman, 2012; Harcourt-Smith et al., 2015). Foot shape and functions are closely related and especially adapted to bipedalism. While examining the foot from a functional perspective, the posterior calcaneus contributes to balance support and impact absorption, the dome-like arch stiffens the foot during weight-bearing and returns energy during push-off in gait, the metatarsals expand the anterior pressure support, and the toes (particularly the hallux) facilitate pushing-off at the end of stance during locomotion (Morton, 1924; Hicks, 1953; Hicks, 1954; Ker et al., 1987; Ward et al., 2011; Fernández et al., 2018; Venkadesan et al., 2020).

While considering the measurement of foot functions, the classification of the foot–ankle complex has been presented using several popular metrics. A commonly employed model in clinics was based on the “Rootian theory” (Root model), also known as the “subtalar joint neutral theory” (Root et al., 1977). This model uses static measurement of the subtalar joint neutral position to predict dynamic functions and prescribe orthotics for treatment. However, this model has been challenged recently for poor (low) correlation with dynamic functions (Jarvis et al., 2017) and concerns of reliability and validity (Harradine et al., 2018). The technique of classifying foot posture types has been reported using visual inspection, anthropometric measurement, footprint analysis, and radiographic assessment for rearfoot, midfoot, and forefoot (Razeghi and Batt, 2002). These include measurement of the arch height, longitudinal arch angle, navicular drop and drift, artificial intelligence and arch angle, and radiographic evaluation of calcaneal inclination angle, height–length ratio, calcaneal–first metatarsal angle, and rearfoot–forefoot angle. The foot posture index (FPI) is another popularly employed metric to define pronated, neutral, or supinated feet *via* anatomical palpation and structural observation (Redmond et al., 2006). In terms of dynamic conditions, a recent minimal markerset model was proposed for navicular position measurement and validated for relating foot postures and functions with accurate intraday reliability (Eichelberger et al., 2018).

In the current literature, several excellent studies have reviewed the relationship of foot shape, foot posture, and foot biomechanical function. Shoe-wearing habits, pathology, or external factors have also played contributing roles in foot shape, posture, muscle and tendon morphology, and bone alignment (Cavanagh et al., 1997; Johnson et al., 2015; Xiang et al., 2018; Garofolini and Taylor, 2019). Specifically, a 10-week transition into running with minimalist shoes found an increased cross-sectional area of abductor hallucis by 10.6% (Johnson et al., 2015). This has confirmed that tissue morphology is related with shoe-wearing habits of minimalist shoes, motion control shoes, and neutral shoes (Zhang X. et al., 2018). Decreased hallux abductus angle (measured from X-ray images) and hallux angle (measured with footprints) have been reported with centrally shifted plantar pressure and reduced medial metatarsals stress followed by a 12-week minimalist shoe intervention for mild hallux valgus (Xiang et al., 2018; Xiang et al., 2022b).

In particular, the foot type and foot disorders are strongly associated with the foot arch difference, classified as pes cavus (high arch), pes rectus (normal arch), and pes planus (flat arch) (Hillstrom et al., 2013; Mootanah et al., 2013). Pes planus feet have been associated with hammer toes and overlapping toes, while pes rectus and pes cavus have not been associated with any foot disorders (Hagedorn et al., 2013). Furthermore, decreased thickness and area in the intrinsic muscles (such as abductor hallucis, flexor hallucis brevis, and peroneus longus and brevis), plantar fascia, and Achilles tendon, while increased thickness and area in the extrinsic muscles (flexor digitorum and flexor hallucis longus) have been found in pes planus, which may implicate the compensatory adaptation for the altered foot structure (Angin et al., 2014, Angin et al., 2018; Crofts et al., 2014; Murley et al., 2014). However, these measurements or analyses were conducted under static conditions.

During walking (dynamic conditions), cavus (high arch) feet presented increased frontal and transverse motion in the rearfoot, while planus (flat arch) feet showed a reduced frontal range of motion in the midfoot (Buldt et al., 2015a). The foot posture index (FPI) showed a stronger correlation with intersegmental kinematics than did the other measurements of the artificial intelligence, navicular height, and dorsal arch height (Buldt et al., 2015b). Additionally, planus feet showed greater activation of the tibialis anterior but less activation of peroneus longus during the initial contact of stance and increased tibialis posterior but decreased peroneus longus activities during midstance and the push-off phase (Murley et al., 2009b). It has been further reported that increased foot inversion and muscle activation were present in pronated feet with less evertor activation (Murley et al., 2009a).

Alteration of shape or posture in the foot, functioning as the primary interface with the external surroundings, may lead to the realignment of the kinetic chain. Foot pronation often showed an everted rearfoot and arch drop in the foot–ankle complex, further inducing internally rotated tibia, proven with a continuous vector coding analysis technique (Rodrigues et al., 2015), and mobile patellar and anterior knee pain (Clifford et al., 2020; Yu et al., 2021) in the knee complex. During the changes in the kinetic chain, the pronated foot posture was further reported to be associated with medial tibia stress injury (Neal et al., 2014), increased subtalar motion, leg stiffness, tibial shock (Hollander et al., 2019), and patellofemoral pain (Selfe et al., 2016) during running. While the posture alteration is not strongly related to lower back pain, apart from females in whom pronated foot showed back pain (Menz et al., 2013). Pronated feet have been strongly associated with hallux valgus and overlapping toes, while supinated feet have shown less association (Hagedorn et al., 2013).

In terms of foot postures and shape in the foot segments, particularly when analyzing the functions of the forefoot *in vivo*, a longer first metatarsal size, rounder first metatarsal head, and greater first metatarsophalangeal joint angle were associated with hallux valgus deformity (Nix et al., 2012). Computational modeling of the foot (Morales-Orcajo et al., 2016) revealed a higher concentrated von Mises stress at the metatarsals and higher contact pressure in the first metatarsophalangeal joint (Zhang et al., 2018c). Furthermore, the diabetic foot presented toe deformation with focalized pressure at the hallux (Lu et al., 2015). Habitually, barefoot populations with increased hallux spacing have been associated with active gripping function (Lambrinudi, 1932; Wallden, 2016), which expands the supporting area in the forefoot (Mei et al., 2015a; Mei et al., 2015b; Shu et al., 2015; Wang et al., 2016) and higher medial longitudinal arch in the barefoot children’s cohorts (Hollander et al., 2017). A nontypical foot shape, such as the bound foot in Chinese women, presents dislocated toes, extreme high arch, limited ankle range of motion, and concentrated loading in the heel (Gu et al., 2015; Zhang et al., 2018b).

Recently, there has been a focus on distance running activities (which is in contrast to static measures or walking) and population-based modeling using large data sets and statistical shape methods (Fernandez et al., 2016; Fernandez et al., 2019). In this study, we first aimed to review and discuss the changes in foot morphology, shape, and posture during dynamic activities, using running as an example. Second, we examined the statistical shape modeling (SSM) of the foot using population-based approaches and reviewed the association between shape, posture, and biomechanics to understand potential injury. Lastly, we summed up the current advances and techniques in analyzing the foot shape, posture, and biomechanical functions and provided several perspective suggestions for consideration in the foot–ankle health-related research.

## Foot morphology, shape, and posture in running activities

### Evaluation of foot morphology, shape, and posture

**FIGURE 1:**
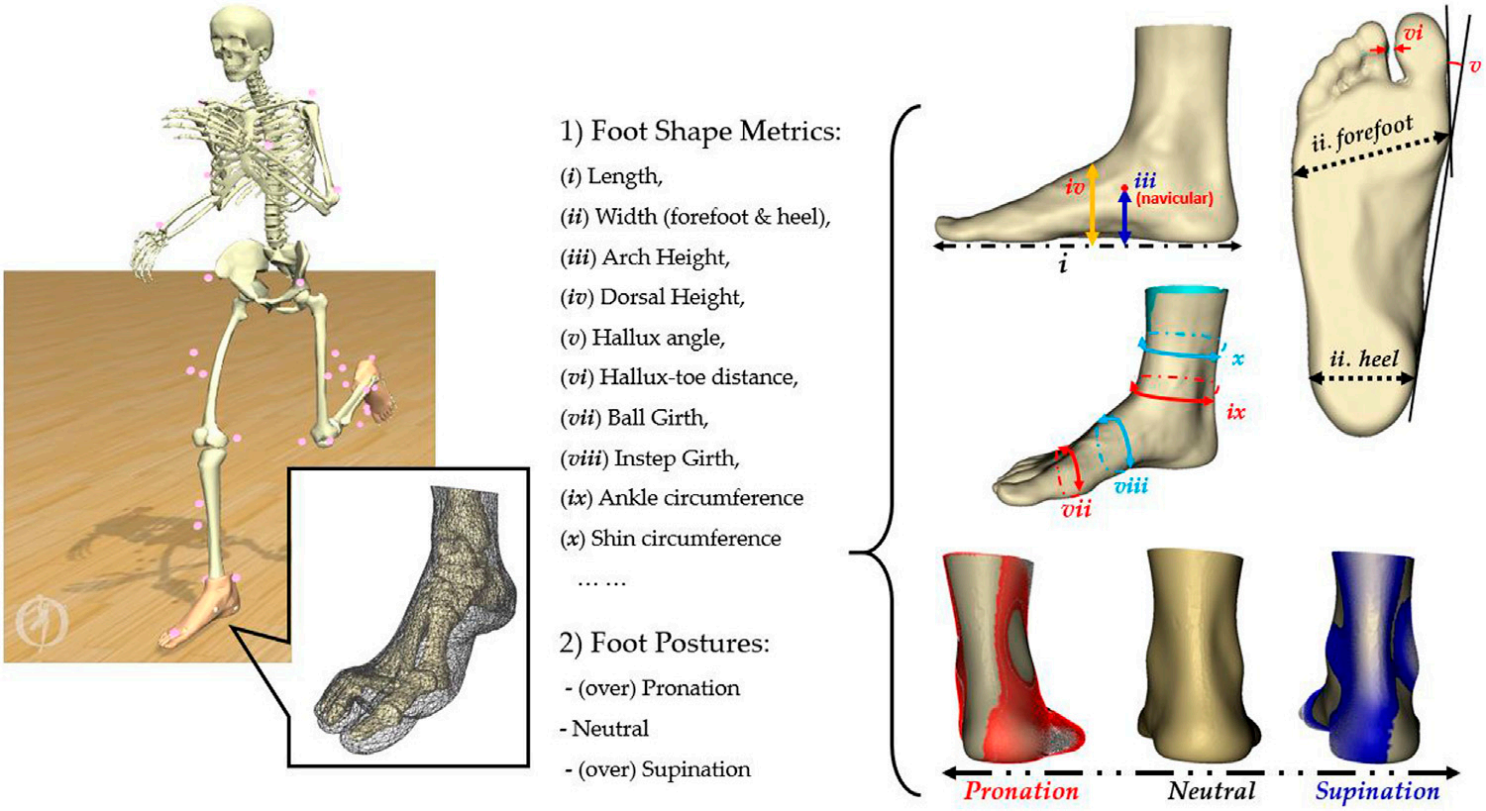
An illustration of popular foot shape metrics and foot postures with highlighted variation of pronation (red regions) and supination (blue regions) from the posterior view.

**FIGURE 2:**
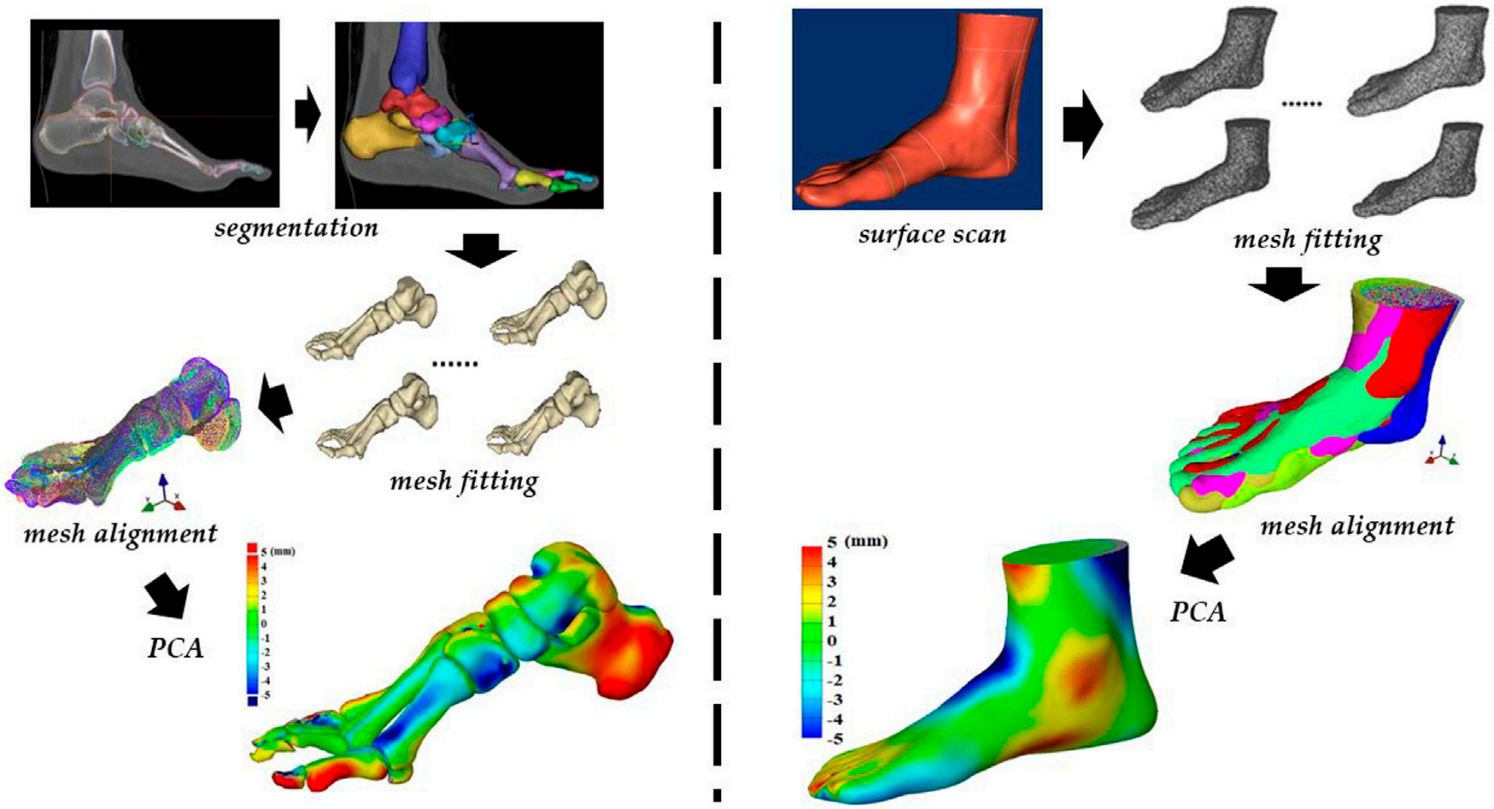
An outline of workflow in the statistical shape modeling (SSM) of foot bones (left) and surface (right) shapes. Steps of segmentation (bone) or surface scan (surface), mesh fitting, mesh alignment, and PCA.

Changes in tissue morphology, bone shape, and posture of the foot influence the biomechanics of the lower extremity. For example, foot types (mainly, normal, planus, and cavus feet) (Buldt et al., 2015a), foot posture (mainly, normal, pronated, and supinated feet) (Hollander et al., 2019), toe morphology (Mei et al., 2015a), hallux valgus (Hannah et al., 2016), and manipulated forefoot shapes (abducted hallux *versus* adducted hallux) (Mei et al., 2016; Xiang et al., 2020a; Xiang et al., 2020b) have been previously reported in the literature. Pathological conditions, such as diabetic-related foot deformities (Guiotto et al., 2013; Lu et al., 2015) can also influence the biomechanics. Figure 1 outlines the most common popular shape metrics (Figure 1) and postures in the foot (Figure 1 and Figure 2) with highlighted regions of variations in pronation (blue) and supination (red) when compared to those of the neutral foot posture. The highlights in pronated and supinated foot postures are modes generated from the principal component analysis (PCA) in the statistical shape modeling (SSM) using an open-source Musculoskeletal Atlas Project (GIAS2 package) developed at the Auckland Bioengineering Institute (Zhang et al., 2014). The SSM typically refers to the techniques of describing the characteristics of deformable objects with different shape features, presenting mean shape (appearance) and key variations in these object groups (Sarkalkan et al., 2014; Mei et al., 2021a).

The current measurement of foot morphology, shape, and posture using 2D footprints, anthropometric measurements, anatomical palpation, 3D surface scanning, ultrasound imaging, computed tomography (CT), or magnetic resonance imaging (MRI) are mainly under static (non-weight or weight-bearing) conditions (Razeghi and Batt, 2002; Redmond et al., 2006; Crofts et al., 2014). However, the foot may present different postures, shapes, and morphologies during dynamic activities (short and long term). Prolonged repetitive impact on bones and soft tissue can alter the shape, biomechanical response, and adaptation (Garofolini and Taylor, 2019). In particular, increased muscle volume and cross-sectional area of foot muscles (abductor halluces, flexor digitorum brevis, and abductor digiti minimi) and bone density (calcaneus) were concluded in a recent review study (Garofolini and Taylor, 2019), which may rely on training volume and experience.

Long-distance running, or endurance running, is a key evolutionary skill presented by bipeds in addition to walking (Bramble and Lieberman, 2004). Long-distance running has gained global popularity with increased marathon participation, particularly in amateurs. However, the increased participation is associated with higher risks of running-related injury (Hulme et al., 2017).

The arch (either high arch or flat foot) in the midfoot region is commonly used to classify and categorize the foot posture in foot biomechanics. Specifically, it has been quantified using the 2D footprint artificial intelligence (AI) and classified as high arch (AI < 0.21), normal arch (0.21 < AI < 0.26), and low arch (AI > 0.26) (Cavanagh and Rodgers, 1987). This metric has been used during static and dynamic measurements (Scholz et al., 2017) and is sensitive to forefoot and midfoot plantar pressures during walking and running (Mei et al., 2020). The lower arch has been associated with increased hallux and medial mid-foot pressures and reduced medial forefoot pressures (Jonely et al., 2011). Increased reported ankle and knee pain have also been associated with lower foot arch (Riskowski et al., 2013), whereas the high arch is primarily associated with ankle pain only (Riskowski et al., 2013). Altered frontal and transverse rearfoot motion and reduced midfoot motion during initial contact are associated with high arch feet, while reduced midfoot motion during pre-swing is associated with low arch feet (Buldt et al., 2015a). While the foot arch is a useful measure in adults, there is a caveat that it might be a less useful measurement in developing and overweight individuals. For example, the dynamic artificial intelligence was not associated with any kinematics, kinetics, or spatiotemporal information in children except for a greater external foot rotation associated with a lower arch (Hollander et al., 2018b). The artificial intelligence is also influenced by training levels and running experience (Fascione et al., 2009) despite the biomechanical evaluation being not reported. Overweight adults (with high BMI) showed pronated and flatter feet with reduced ankle inversion–eversion motion and higher plantar loading underneath the foot (Butterworth et al., 2015), which led to uncertainty of either the change of foot structure or increased weight contributing to a greater plantar loading.

### Relationship between morphology, shape, and posture with running biomechanics

Running biomechanics influences the foot structure. Following long-distance running, foot arch and dorsal height reduce over a week and may take more than a week to return to pre-run profiles and has been linked with increased plantar loadings in the medial foot (metatarsals and arch) (Fukano and Iso, 2016; Fukano et al., 2018; Mei et al., 2018). These changes could affect the perceived comfort of the running footwear and contribute to running-related injuries (Cowley and Marsden, 2013; Hollander et al., 2018a). Following a running-induced fatigue intervention, plantar pressure has been reported to redistribute in the lateral metatarsals of flat arch feet and the medial metatarsals of high arch feet (Anbarian and Esmaeili, 2016). Following long-distance running, a pronated foot posture, reduced arch height, and increase in medial plantar pressure have been reported (Nagel et al., 2008; Schlee et al., 2009; Cowley and Marsden, 2013). The FPI (Redmond et al., 2006) following long-distance running has been moderately correlated with knee and ankle joint loads using the musculoskeletal model (Mei et al., 2019). Furthermore, runners with asymptomatic overpronated feet have larger abductor hallucis and flexor digitorum brevis and longus but smaller abductor digiti minimi, and peak eversion in the rearfoot and peak supination in the forefoot (Zhang et al., 2017). While, symptomatic overpronated runners have shown a smaller cross-sectional area in the flexor digitorum longus and abductor hallucis and thinner peroneus and abductor hallucis than those asymptomatic pronated runners, implying the training of intrinsic foot muscle for the possible prevention of injuries (Zhang et al., 2019).

### Associations between foot pronation and injury mechanisms

Foot types (mainly, normal, planus, and cavus feet) and postures (mainly, normal, pronated, and supinated feet) have been reported to be associated with running-related injuries (Pérez-Morcillo et al., 2019), particularly the tibia stress under extreme foot types (Barnes et al., 2008), medial tibia stress, and patellofemoral pain with foot pronation (Lun et al., 2004; Neal et al., 2014; Selfe et al., 2016). The hip joint loading was found to be increased, whereas only a moderate correlation with foot pronation and ankle and knee loadings was reported (Mei et al., 2019).

This subsection is focused on foot pronation and potential contribution to running-related injuries, taking distance running as a typical example. Overpronated feet have been implicated in developing overuse injuries, despite there being no scientific evidence that overpronated feet are associated with the diagnosis of injuries or diseases. This misconception exists possibly because altered foot postures have often been observed in people who have musculoskeletal injuries, dysfunctions of the lower limb, and lower back pain (Levinger et al., 2010; McWilliams et al., 2010; Sharma et al., 2010; Menz et al., 2013; Arnold et al., 2019).

Pronation/supination are important biomechanical functions in gait, and a certain extent of natural pronation/supinations is required as a shock absorber during the early stance phase and as a rigid lever to push forward during the terminal stance phase (Hetsroni et al., 2008). During repetitive movements such as long-distance running, however, the high volume of impact forces during the early stance phase may lead to overpronation (Mei et al., 2019) by flattening the foot with arch collapses and transferring the foot eversion into the internal rotation of the tibia. There has been a belief that this overpronation possibly leads to overuse injuries by disrupting the coupling mechanism of the lower limb alignment as the gait may be compromised and adding the additional strain of the foot/ankle complex (Subotnick, 1985). Pronation is also a passive force that occurs within the initial heel strike during walking and running. That is, there would be less muscular control when the foot is overpronated, resulting in a lack of normal distribution of excessive force and instability of the foot/ankle (Subotnick, 1985). This patho-mechanical alteration of foot posture, therefore, has been proposed to cause foot diseases such as plantar fasciitis (Golightly et al., 2014), osteoarthritis (Reilly et al., 2009; Lithgow et al., 2020), metatarsalgia (Eustace et al., 1993), and stress fractures of the lower limb (Lysholm and Wiklander, 1987).

Overpronated feet, specifically at the subtalar joint, have been proposed to develop deterioration of the lower limb joint by disrupting normal alignment with external rotation of the tibia and calcaneal inversion which are not in the normal direction (Tiberio, 1987; Hintermann and Nigg, 1998). A previous study also showed that people with medial compartment knee osteoarthritis revealed a more pronated foot than healthy controls, possibly due in part to genu varum malalignment of the knee, which causes compensatory overpronation of the pronated foot (Levinger et al., 2010). Pronation with adduction of the talus with calcaneus eversion results in a greater compressive force in the medial midfoot, and this idea has been supported by several studies, reporting that people who have been diagnosed with midfoot osteoarthritis have a more pronated foot posture (Menz et al., 2010; Arnold et al., 2019; Arnold et al., 2021; Lithgow et al., 2020). Older adults, who were diagnosed with radiographic osteoarthritis of the talonavicular joint and navicular–first cuneiform joint, presented flatter feet with greater loading of the midfoot during walking (Menz et al., 2010). This mechanism has also been supported by a cadaver study, reporting a greater compression force at the dorsal talonavicular joint simulating a flattening foot (Kitaoka et al., 1996).

However, while numerous studies have observed pronation/supination of the foot in people who have lower limb diseases, there is no scientific evidence supporting the causal relationship between the alteration of foot posture and diagnosis of injuries or diseases. A recent study on knee osteoarthritis with foot posture also put forward the question of foot postural changes leading to injuries or injuries resulting in posture changes (Al-Bayati et al., 2018). A comprehensive cohort study of running-related injuries in a large population of 1,680 runners reported that there was no significant association between anthropometric outcomes (e.g., high/low arch and rearfoot valgus) and risk factors of running-related injuries (Water et al., 1960). Similarly, a 1-year epidemiological prospective cohort study of 927 novice runners reported that foot pronation is not associated with an increased risk of running-related injuries (Nielsen et al., 2014). A systematic review study revealed a small effect between foot pronation and the risk of medial tibial stress syndrome, suggesting that foot pronation may not be directly associated with the foot injury (Neal et al., 2014). Furthermore, a more recent radiologic study reported that significant ankle kinematic changes associated with supination of the foot were not related to the diagnosis of diseases (Kim et al., 2019a; Kim et al., 2021). This study interestingly observed that novice runners, who did not show ankle kinematic changes after mid-distance barefoot running, revealed early indications of cartilage degeneration or deteriorating effects by increasing the T2 relaxation time in MRI-derived T2 maps. However, runners who showed a supinated foot type after mid-distance running did not change their T2 value on MRI. This study, therefore, suggests that supinated feet or significant ankle kinematic changes are less likely to develop foot/ankle injuries.

The idea of our understanding of the association between pronation/supination and running-related injuries is still not clear and no consensus has been reached on the foot posture with injuries (Nigg et al., 2019). Thus, to draw a conclusive result, we may require additional information such as the integrated longitudinal arch angle and Achilles tendon angle proposed for the determination of foot postures and biomechanics during walking and running (Limeres et al., 2019; Behling and Nigg, 2020). Due to multifactorial parameters being included for analysis, multivariate statistical models, such as principal component analysis (Limeres et al., 2019; Behling and Nigg, 2020), partial least square regression (Mei et al., 2020), and other nonlinear statistical models, are recommended to investigate the potential correlation.

Numerous foot imaging modalities have been adopted to capture the foot posture *via* shape which include the 3D plantar surface (Kimura et al., 2008; Thabet et al., 2014), dorsal surface (Blenkinsopp et al., 2012), and whole foot (Boppana and Anderson, 2019). Techniques of tracking *in vivo* foot motion have been previously developed and validated using makers in a biplane fluoroscopy system (Iaquinto et al., 2014), and midfoot postures have been analyzed to evaluate the longitudinal arch angle under conditions of barefoot (∼127.5°), footwear (∼130°), and orthoses (∼131°) (Mannen et al., 2018). However, challenges of multiaxial motion in the ankle complexity of tibiotalar and subtalar joints were found (Canton et al., 2020). The development of *in vivo* bone shapes in the foot has laid the foundation of classifying foot types from 3D perspectives (Ledoux et al., 2006). Recent attempts have been made to associate static foot bone images from 3D CT with 2D biplanar video-radiography images *in vivo* (Maharaj et al., 2020) and the development of the multi-segmental foot musculoskeletal model (Malaquias et al., 2017; Maharaj et al., 2021), but the cost of obtaining subject-specific bone geometry makes this method less translatable for practical use. Population-based modeling using ‘big data’ may provide an alternative for rapidly creating foot geometries from limited data [such as the popularly employed statistical shape modeling of functional foot bones (Grant et al., 2020)] and establishing relationships between form and function at the population level.

## Population-based modeling of shapes

### Statistical shape modeling with principal component analysis

Statistical shape modeling (SSM) is a reduction technique that can be used to identify independent (orthogonal) geometrical features of a set of similar shapes and rank them (Cootes et al., 1995). The principal component analysis (PCA) is the most popular method to reduce dimensionality and computes the mean shape and orthogonal shape variations (modes) (King and Eckersley, 2019). This technique has been used widely in the biomechanics and anatomy space (Ambellan et al., 2019; Audenaert et al., 2019; Fernandez et al., 2019; Vallabh et al., 2019; Wang and Fernandez, 2020; Yeung et al., 2020). Applications include clinical medical image analysis (Heimann and Meinzer, 2009) and surgery and design of orthopedic implants (Sarkalkan et al., 2014).

To introduce one of the SSM technique, we took musculoskeletal modeling software GIAS2 (Zhang et al., 2014) (https://pypi.org/project/gias2/) being developed at the Auckland Bioengineering Institute as an example. Figure 2 demonstrates the workflow in the SSM of the foot surface shape (right side) and bone shape (left side) being employed from our research group. The steps include shape mesh segmentation (bone shape) or surface scan (surface shape), mesh fitting and alignment, and the principal component analysis (PCA) to compute the mean shape and key modes of variation.

Population-based approaches for understanding the foot have been reported in osteoarthritis populations (Trivedi et al., 2010), for the footwear type and foot discomfort in females (Dufour et al., 2009), flat arch with ankle pain and high arch with knee pain (Riskowski et al., 2013), and hallux valgus with foot pain (Dufour et al., 2014). Recent advances in foot imaging technology have enabled easy access and increased availability of 3D foot scanning systems (Telfer and Woodburn, 2010), providing increased foot morphology data for the study of shape variations, especially in populations of different ethnicities (Mei et al., 2021b; Mei et al., 2021a). A recent study highlighted published 1.2 million foot shape data on the features of populations from North America, Europe, and Asia and reported the distribution of geometrical metrics (such as length, width, and height) between males and females (Jurca et al., 2019). Apart from the measurements from 3D scanning technologies, the PCA on the 3D shape is one common and useful approach to extract meaningful information from these data (Mei et al., 2021a).

The SSM of the calcaneus and talus bones in the rearfoot revealed a smaller size in females but less asymmetry in both genders (Audenaert et al., 2019), specifically in the length and height of calcaneus and talus articular surface (Tümer et al., 2019a). In contrast to the normal foot, the calcaneus shape presents decreased height and increased length in high arch feet and increased posterior mass in the talus of flat arch feet (Moore et al., 2019). A statistical shape model of the foot surface reported variations in arch height, ball (metatarsophalangeal joint) width, toe distance, hallux orientation (valgus–varus), and toe length (Stanković et al., 2018). A higher BMI has been related to greater ankle width, Achilles tendon size, and width. Age was associated with heel width, Achilles tendon size, and hallux orientation. Gender was linked to ankle width, Achilles tendon size, and heel width. Classifying problematic feet using surface morphology has been reported which included hallux valgus, pes planus, and pes cavus profiles (Stanković et al., 2020).

A flat talar contact surface has been associated with chronic ankle instability (Tümer et al., 2019b). This flat talar surface may be associated with adaptation to constraining footwear on the basis of comparisons with the talus shape from archaeological records (Sorrentino et al., 2020). High arch feet have a more posteriorly positioned navicular tuberosity than normal arch feet (Moore et al., 2019). In the forefoot, metatarsal shapes are associated with foot types (high arch, normal, and flat arch) and bones vary in size (Telfer et al., 2017). Specifically, the flat arch foot has a reduced cross-sectional metatarsal area, and the first and fourth metatarsal sizes are linked to gender, with females presenting smaller sizes. Furthermore, the first metatarsal could be accurately reconstructed from sparse landmarks based on the SSM (Grant et al., 2020), which has the potential for rapid development of the musculoskeletal foot models (Grant et al., 2020).

### Recent techniques to correlate shape with function in the foot

One of the challenges with the SSM is creating a population of topologically consistent geometries for the principal component analysis. This problem has been addressed by using the ‘free-form’ deformation technique (Fernandez et al., 2004), where a generic geometry is morphed to different data (‘host mesh’). This has been demonstrated for the diabetic foot (Fernandez et al., 2012) and gout foot (Dalbeth et al., 2015) and, recently, for investigating ankle pressure in barefoot runners (Kim et al., 2019b). This technique has been used to check the similarity of foot shapes (Mochimaru et al., 2000), which are combined with plantar pressures for the application of footwear design (Kim et al., 2007). Another consideration which should be noted is that the prediction of four-dimensional (3D shapes varying over time) foot shapes become plausible from integrating multidisciplinary advanced statistics, artificial intelligence (AI), depth camera, and object detection techniques (Boppana and Anderson, 2019; Boppana and Anderson, 2021). The development of this technology may become a great improvement in shape changes in real time and provide an option of considering footwear and orthotics fit from a dynamic and functional perspective.

Recently, multivariate machine learning (partial least squares) regression models were first developed to correlate key shape metrics (artificial intelligence and hallux–toe distance) with walking (R-square values of 0.763 and 0.788) and running (R-square values of 0.786 and 0.789) plantar pressures using habitually barefoot and shod populations (Mei et al., 2020), showing a prediction accuracy of around 80%. Further sensitivity analysis reported that the forefoot shape metric (hallux–toe distance) was associated with medial forefoot pressures, especially during walking in habitually barefoot populations. Also, the midfoot shape metric (artificial intelligence) was associated with lateral forefoot pressures during walking in both habitually barefoot and shod populations. An improved predictive statistical model (support vector machine) also showed an increased prediction accuracy on the basis of the same data set (Xiang et al., 2022a). Rapid prediction of foot function from easily measured foot shape metrics may become the norm and extend into clinical diagnosis of foot pathologies and customized footwear development.

## Conclusion and future perspectives

This review has explored known associations between foot shape, posture, and foot conditions. The foot artificial intelligence was found to be a useful metric of foot posture, which can be measured quickly and associated with motion and joint pain in adults; however, it may be less useful in special population groups, such as developing and obese individuals. The foot posture index to classify feet as pronated, neutral, and supinated is a useful approach under both static and dynamic conditions, but care must be taken when using it to associate pronation with injury risk. While recent studies have associated foot posture with joint loading, more information, such as factors of Achilles tendon angle and longitudinal arch angle, is needed to elucidate risk from normal function. With increasing imaging technology and database sharing, there is an opportunity to apply statistical shape modeling methods to derive learnt information from ‘big data’ and use this to make a population-based inference and fill in missing data from personalized information. Foot bone shapes and tissue morphology have been associated with pathology, gender, age, and height and may help develop rapid population-specific foot classifiers. A popular topic, barefoot running, was investigated, and it was shown that the forefoot toe shape influenced forefoot plantar pressure in habitually barefoot runners only, while the arch shape influenced plantar pressure in any population. This may play a role in footwear design.

Based on findings from the current review and potential gaps in the literature, future studies may consider the following topics *via* 1) tracking the internal foot motion during dynamic activities *via* biplanar fluoroscopy and multi-segmental models. With reported increased measuring accuracy, mapping the biplanar 2D motion to 3D shape motion and statistical shape modeling *in vivo* would assist the validation of finite element modeling and reveal the tissue variation, ligament strain, and cartilage loadings; 2) implementing different multivariate (support-vector) machine learning or convolutional neural network (CNN) algorithm to address potential nonlinear correlation scenarios in foot mechanics with shape or posture metrics; 3) standardizing data sets with synchronized IMU data for rapid prediction of instant mechanics, load accumulation, injury risks, and adaptation in the tissues and bones of the foot and correlating with foot shape; 4) analyzing the dynamic foot shape and posture *via* marker-less depth camera and real-time processing techniques under real-life scenarios for precise evaluation of clinical foot conditions and performance-fit footwear development.
